# Active layer and permafrost microbial community coalescence increases soil activity and diversity in mixed communities compared to permafrost alone

**DOI:** 10.3389/fmicb.2025.1579156

**Published:** 2025-06-05

**Authors:** Stacey J. Doherty, Alison K. Thurston, Robyn A. Barbato

**Affiliations:** United States Army, Engineer Research Development Center, Cold Regions Research and Engineering Laboratory, Hanover, NH, United States

**Keywords:** permafrost, soil mixing, community level physiological profiling, community coalescence, microbial community, Alaska

## Abstract

Permafrost is experiencing rapid degradation due to climate warming. Microbial communities undergo significant compositional and functional shifts as permafrost thaws. Dispersal of microbial communities from the seasonally-thawed active layer soil into newly thawed permafrost may influence community assembly and increase carbon release from soils. We conducted a laboratory soil mixing study to understand how carbon utilization, heterotrophic respiration, and microbial community structure were affected when active layer and permafrost soils were mixed in varying proportions, as what is expected to occur when the terrain thaws. Active layer soil and permafrost collected from two sites in Alaska were mixed in five different ratios and incubated for 100 days at 10°C to reflect current maximum surface soil temperatures at these sites. Respiration rates were highest in the 100% active layer soils, averaging 19.8 μg C-CO_2_ g^–1^ dry soil d^–1^ across both sites, and decreased linearly as the ratio of permafrost increased. Mixing of the two soil layers resulted in utilization of a more diverse group of carbon substrates compared to permafrost alone. Additionally, combining active layer and permafrost soils increased microbial diversity and resulted in communities resembling those from the active layer when soils were mixed in equal ratios. Microbial communities of the experimentally mixed soils did not resemble those collected from the transition zone. Understanding the effects of active layer-permafrost mixing on functional potential and soil organic matter decomposition will improve predictions of carbon-climate feedbacks as permafrost thaws in these regions.

## Graphical Abstract

The influence of soil mixing on microbial community functional activity and diversity. In the field environment, seasonally thawed AL soil contains a more diverse and active microbially community compared to permanently frozen PF below. Experimentally mixing these soil layers results in an increase in soil functional activity and diversity when a small amount of AL soil is added to PF. Conversely, when a relatively small amount of PF is added to AL, there is not a significant change in functional activity compared to AL alone. Created in BioRender. Doherty (2024) BioRender.com/h41c049. 
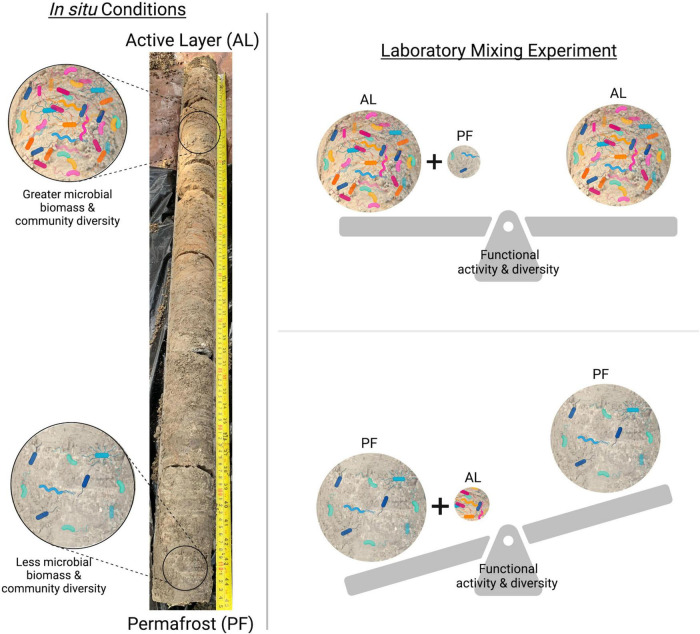

## 1 Introduction

Permafrost—perennially frozen ground—is widespread in the circumpolar north with current estimates approximating 15% of the northern hemisphere is underlain by permafrost (PF) (Obu et al., 2019). Above the PF is a seasonally thawed active layer (AL) soil that warms in the summer and freezes in the winter. PF contains approximately 1,700 Pg of soil carbon which is almost twice as much carbon than in our atmosphere (Schuur et al., 2013). Climate change is resulting in rapid PF thaw leaving this soil carbon vulnerable to microbial decomposition and release as greenhouse gases (Schuur et al., 2022). There remains uncertainty in the projections of carbon release due to climate warming in various Earth system models and climate warming feedbacks (Schuur et al., 2022).

Soil microbial communities are important drivers of global carbon cycling. Previous studies have shown PF microbiomes are site specific (Barbato et al., 2022; Waldrop et al., 2023), distinct from those found in the AL (Mackelprang et al., 2011; Monteux et al., 2018; Doherty et al., 2020), and undergo significant shifts to both community composition and functional potential as soils thaw (Mackelprang et al., 2011; Doherty et al., 2020; Barbato et al., 2022). Much of our understanding of PF microbiome shifts upon thaw has resulted from laboratory incubation studies that neglect the potential influence of microbes from the AL soil perched above the PF. Dispersal is likely a key factor influencing microbial community assembly as PF thaws with the AL microbial community contributing to the post-thaw microbiome’s activity and function (Ernakovich et al., 2022). Understanding microbial community dynamics with PF thaw may improve estimates of carbon cycling since microbes are responsible for CO_2_ and CH_4_ release as soil organic matter is decomposed.

The concept of community coalescence describes situations where two or more distinct communities and their local environments combine (Rillig et al., 2015; Liu and Salles, 2024). Thawing PF creates opportunities for microbial coalescence events through cryoturbation, drainage of thaw ponds, and ground slumping. The relative size (i.e., mixing ratios) of the interacting communities, their respective environments, and community diversity are important factors influencing the outcomes of community coalescence (Rillig et al., 2015; Diaz-Colunga et al., 2022; Huet et al., 2023). For instance, the small addition of one community to a larger community in its native environment may give the latter a competitive advantage (Rillig et al., 2015). AL soils are generally more taxonomically and functionally diverse than PF soils (Ernakovich and Wallenstein, 2015; Tripathi et al., 2018) and may prevail upon mixing with PF. Furthermore, we expect AL and PF microbial communities to have some unique trait space given major differences in their environments (e.g., constant frozen conditions in PF, aerobic vs anaerobic, freeze-thaw tolerance). When two communities with distinct trait spaces combine, it is theorized that functional activity will be altered; however, when two communities are similar in trait space, little change will be observed in community function (Rillig et al., 2015, 2016). Questions remain if coalesced communities will exhibit broader functional potential compared to isolated communities since new functions could be introduced to the metacommunity through merging of local communities (Rillig et al., 2015). If this were the case for mixed AL and PF communities, there could be significant carbon feedbacks that are currently unaccounted for if mixed communities were better suited to decompose soil organic matter upon thaw.

We tested whether mixing of AL and PF soils would affect carbon utilization and microbial community coalescence when PF thaws. We hypothesized that even a small portion of AL soil added to PF would increase carbon utilization activity and diversity, but when a small portion of PF was added to AL soil, there would be little effect on carbon utilization because AL soils generally contain greater microbial biomass and diversity compared to PF. We also compared results from the experimentally mixed soils to those collected from the transition zone (TZ) where AL and PF soils meet *in situ*, hypothesizing that the TZ might have similar activity and microbial diversity to our mixed samples. To test these hypotheses, we measured heterotrophic respiration for 100 days after mixing AL and PF soils in varying proportions. At the end of the incubation period, we measured community-level physiological profiling to understand how carbon utilization (i.e., functional activity) was affected by mixing. We also investigated microbial community composition to determine if mixed soil communities more closely resembled the dominant soil added and the degree at which community coalescence would play a role in the mixed soils.

## 2 Materials and methods

### 2.1 Soil collection and preparation

Two soil cores spanning AL to PF were collected from two locations in Alaska. The first core was collected in October 2019 to a depth of 140 cm at a thermokarst site near the Toolik Lake Field Station (TK1; 68.649265, −149.509637). The depth of the seasonally thawed AL was 57 cm in October 2019. The TK1 site is situated in a continuous PF zone with thermokarst features. The second core was collected in March 2023 above the Cold Regions Research and Engineering Laboratory (CRREL) Permafrost Tunnel Research Facility in Fox, AK (APT; 64.9507, −147.6200) to a depth of 124 cm. The AL depth at this site is typically 40–50 cm in summer. The APT site is located in a boreal forest with black spruce, Sphagnum moss, and dwarf birch.

At TK1, the thawed active layer was removed using a shovel cleaned with 70% isopropanol, DNAaway™ (Molecular BioProducts, San Diego, CA), and RNAse Away™ (Molecular BioProducts, San Diego, CA) and placed in a sterile Whirlpak™ bag (Nasco Sampling, Chicago, IL). APT was collected in March when the ground was still frozen. At each site, frozen active layer and permafrost soils were collected using a SIPRE corer (8 cm inner diameter, Jon’s Machine Shop, Fairbanks, AK) fitted with a gas-powered motor. All personnel wore Tyvek suits and nitrile gloves when handling the soils. The barrel of the SIPRE corer was cleaned with 70% isopropanol, DNAaway™ and RNAse Away™. The depth of each core segment was noted at the time of sampling in order to reconstruct the depth profile back in the lab. The samples were kept frozen during transport to CRREL in Hanover, NH where the TK1 core was stored at −80°C and the APT core was stored at −10°C. TK1 was stored at −80°C to limit microbial compositional changes pre-experimentation since it was collected 4 years earlier than APT.

Since the frozen soils were not collected aseptically in the field, samples were processed in a −10°C cold room to remove the outside material. The outside of each core was scraped twice using a microtome blade cleaned with 70% ethanol (Barbato et al., 2016). The core was then secured in a bench vice lined with aluminum foil that was sterilized by baking in a muffle furnace at 450°C for 4 h. Both ends of the core that were not fully scraped clean were removed by scaring the core with a heat-sterilized wire saw then loosened with a 70% ethanol cleaned chisel and mallet. Bulk samples were placed in new sterile Whirlpak™ bags and returned to original storage temperature until thawed for the incubation study.

### 2.2 Soil incubation and heterotrophic respiration

Soil samples were removed from the freezer and placed in a 4 °C refrigerator for 2 days to thaw in their individual containers. Samples were then pooled separately for each site into active layer (AL), transition zone (TZ), and permafrost (PF) based on the AL depth measurements collected at the sites during the time of sample collection. For the TK1 site, samples from 0 to 35 cm were pooled as the active layer, 35–57 cm as the transition zone, and 57–140 cm for the permafrost. For the APT site, active layer soil was pooled from 0 to 45 cm depths, the transition zone ranged from 45 to 60 cm, and permafrost included samples from 60 to 124 cm depths.

Pooled samples were homogenized by mixing soils by hand in a sterile bag and placed in a 10°C incubator (Yamato Scientific America Inc., Santa Clara, CA) for 6 days to preincubate and allow for microbial mixing within each soil layer (i.e., AL, TZ, or PF). The APT AL bulk soil was homogenized by briefly blending in a stainless steel blender (Waring Laboratory, Stamford, CT) cleaned with 70% ethanol, DNAaway™, and RNAse Away™ (Molecular BioProducts, San Diego, CA) due to the inability to homogenize the peat soil by hand. Because the thawed PF and TZ samples were water saturated, a sterilized beaker and stir plate were used to keep the soil homogenized while the appropriate mass of soil slurry was pipetted using a serological pipet during microcosm construction.

For the incubation study, AL and PF soils were mixed in five ratios—100% AL, 90% AL + 10% PF, 50% AL + 50% PF, 10% AL + 90% PF, and 100% PF—as well as a 100% TZ sample to represent the active layer-permafrost boundary where the AL and PF soil layers meet in the field. Field-moist soils were used to create batch soils of each mix ratio and construct quadruplicate microcosms containing 20 g dry mass. Soil mixes were based on equivalent dry mass of soil (e.g., each 10% AL + 90% PF microcosm contained 2 g dry AL and 18 g dry PF). Subsamples were taken from only the 100% AL, 100% TZ, and 100% PF samples for baseline assessment (t0) of gravimetric water content (GWC) and microbial community structure and function as determined by amplicon sequencing and EcoPlate™ analysis, respectively.

For each of the 48 microcosms, approximately 45 g fresh mass (equivalent to 20 g dry mass) was transferred to an autoclave-sterilized 250 mL wide mouth glass jar. The jars were sealed with a rubber o-ring, metal lid fitted with two quick connect ports and a threaded cap. Microcosms were placed in a dark 10°C incubator and connected to a closed-loop MicroOxymax Respirometer (Columbus Instruments, Columbus, OH, United States) to monitor heterotrophic respiration (CO_2_) every 12 h throughout the 100 days incubation. Two empty jars were also connected to the respirometer to serve as negative controls for the experiment. Incubation temperature was chosen to reflect maximum summertime surface soil temperatures at APT (unpublished data) (Douglas et al., 2021). Soil respiration rates over the last 108 h of incubation were averaged across replicates to reflect stabilized respiration rates of the microbial communities in each treatment when the slope of the rate was less than 0.005. After 100 days of incubation each microcosm was subsampled for soil properties, DNA, and EcoPlate™ analysis. Subsamples for soil properties were processed immediately, while approximately 5 g of soil was frozen at −80°C for DNA analysis, and 2 g of soil was weighed into a 50 mL conical tube for EcoPlate™ analysis and incubated at 10°C overnight until they were inoculated the following day.

### 2.3 Soil properties

Soil properties were measured at the conclusion of the incubation study. Gravimetric water content was determined by drying soils at 105°C for 24 h and calculating the percent water content on a dry mass basis (Black, 1965). Soil pH was determined by combining soil and deionized water (1:5), shaking for 1 h at 150 rpm, and then left to settle for 10 min (Kabała et al., 2016). Samples were then measured with a Hanna Instruments pH probe (Woonsocket, RI, United States) connected to a SevenEasy S20 pH meter (Mettler Toledo, Columbus, OH, United States). Air-dried samples were sieved to 2 mm, ground to a fine powder using a mortar and pestle, and sent for total carbon (TC) and nitrogen (TN) analysis by the Oregon State University Soil Health Lab. TC and TN were measured by dry combustion on a Vario Macro Cube (Elementar, Langenselbold, Germany).

### 2.4 Biolog EcoPlate™ assay

The Biolog EcoPlate™ (Biolog, Hayward, CA, United States) assay was used to survey metabolic patterns of the mixed communities by measuring carbon substrate utilization. The EcoPlate™ contains 31 carbon substrates that can be categorized into six substrate classes representing amines, amino acids, carbohydrates, carboxylic acids, polymers, and phenolic compounds (Sala et al., 2010; Table 1). For each sample, 2 g of fresh soil was weighed into a sterile 50 mL conical tube and incubated overnight at 10°C. Soil was combined with 18 mL of autoclave-sterilized phosphate buffered saline (1X PBS) and placed horizontally on an orbital shaker at 150 rpm in a 4°C cold room for 1 h. Samples were then placed vertically in the 10°C incubator for 20 min to allow soil particles to settle. To prevent rapid dye saturation, soil dilutions with 1X PBS were performed on mixtures primarily containing active layer and transition zone soils. The 100% AL and 90% AL + 10% PF samples were diluted to 10^–3^, while the 100% TZ and 50% AL + 50% PF samples were diluted to 10^–2^. The 100% PF and 10% AL + 90% PF samples were not diluted further. The supernatant was transferred to a sterile reservoir and 150 μL was pipetted into each well of the EcoPlate™. Plates were measured approximately every 24 h for 40 days using the Biolog OmniLog System (Biolog, Hayward, CA, United States) to capture saturation of the tetrazolium dye for majority of the substrates. Between readings, plates were incubated at 10°C (Yamato Scientific America Inc., Santa Clara, CA, United States) in a closed container with a small beaker of water to help maintain moisture.

**TABLE 1:** Biolog Ecoplate™ substrates grouped by substrate class.

Compound	Substrate class
L-Arginine	Amino acid
L-Asparagine	Amino acid
L-Phenylalanine	Amino acid
L-Serine	Amino acid
β-Hydroxy-Glycyl-L-Glutamic Acid	Amino acid
L-Threonine	Amino acid
Phenylethylamine	Amine
Putrescine	Amine
D-Mannitol	Carbohydrate
Glucose-1-Phosphate	Carbohydrate
D,L-α-Glycerol Phosphate	Carbohydrate
β-Methyl-D-Glucoside	Carbohydrate
D-Galactonic Acid γ-Lactone	Carbohydrate
i-Erythritol	Carbohydrate
D-Xylose	Carbohydrate
N-Acetyl-D-Glucosamine	Carbohydrate
D-Cellobiose	Carbohydrate
α-D-Lactose	Carbohydrate
D-Glucosaminic Acid	Carboxylic acid
D-Malic Acid	Carboxylic acid
Itaconic Acid	Carboxylic acid
Piruvic Acid Methyl Ester	Carboxylic acid
D-Galacturonic Acid	Carboxylic acid
α-Keto Butyric Acid	Carboxylic acid
γ-Amino Butyric Acid	Carboxylic acid
2-Hydroxy Benzoic Acid	Phenolic compound
4-Hydroxy Benzoic Acid	Phenolic compound
Tween 80	Polymer
Tween 40	Polymer
α-Cyclodextrin	Polymer
Glycogen	Polymer

List from Sala et al. (2010).

EcoPlate™ results were analyzed in R version 4.3.2 (R Core Team, 2018). Average well color development (AWCD) was calculated by first subtracting the averaged values from negative control wells from all substrate-amended wells within a plate and then summing values across all substrate wells and dividing by the total number of substrates (Németh et al., 2021). The average AWCD for each mix ratio was plotted over time to assess substrate utilization during growth. Once substrate utilization curves plateaued, final values at day 40 were used to calculate substrate-specific average well color development (SAWCD) and diversity metrics (Németh et al., 2021). Substrate utilization diversity was calculated using the Shannon diversity index to visualize carbon utilization diversity across mix ratios.

### 2.5 Quantitative PCR (qPCR)

Genomic DNA was extracted according to manufacturer protocols using the Qiagen DNeasy PowerSoil Pro Kit with the Qiacube automated DNA extractor (Qiagen, Hilden, Germany). DNA concentrations were quantified using the Qubit 3.0 fluorometer and Quant-iT dsDNA Broad Range Assay Kit (Invitrogen, Carlsbad, CA, United States). qPCR was performed to assess bacterial 16S rRNA gene copy number per sample on a LightCycler 480 System (Roche Molecular Systems, Inc., Indianapolis, IN). The V3 region of the 16S rRNA gene was amplified using the Eub338, 5′-ACT CCT ACG GGA GGC AGC AG-3′ (Lane, 1991) and Eub518 5′- ATT ACC GCG GCT GCT GG-3′ (Muyzer et al., 1993).

Samples were diluted to a DNA concentration of 1.25 ng/μL. Each 20 μL reaction contained 0.5 μM of each primer, 2 μL of template DNA, and 10 μL of the LightCycler 480 SYBR Green I Master (Roche Diagnostics North America). The conditions for qPCR were as follows: a denaturation step of 95°C for 300 s followed by 45 cycles of 95°C for 10 s, 60°C for 20 s, 72°C for 25 s. A melting curve was preformed from 65°C to 97°C. The cycle threshold value was identified using the second derivative maximum method (LightCycler 480 Software, Version 1.5). The gene copy number for each sample was determined using the external calibration curve. Samples were run in duplicate and averaged.

A plasmid standard containing the V3 target region was generated using DNA extracted from *Pseudomonas protegens* (ATCC, Virginia, United States, BAA-447). The amplified DNA was checked on a 1.5% agarose gel. The amplified product was cloned into the pCR 2.1-TOPO vector from the TOPO TA cloning kit (Invitrogen, Waltham, MA, K450001) following the manufacturer’s instructions. Plasmids were recovered from positive transformants using the QIAprep Spin Miniprep Kit (Qiagen, Hilden, Germany, 27106). Plasmid DNA was linearized by performing restriction enzyme digestion with the *Eco*RV-HF enzyme (NEB, Ipswich, MA, R3195T) and the digestion was checked by running the reaction on a 0.8% agarose gel. The DNA concentration was measured using a Qubit fluorometer and the gene copy number per μL of standard was calculated assuming an average molecular weight of 660 g/mol per base pair of DNA. Standard dilutions ranging from 6 × 10^1^ to 6 × 10^8^ 16S rRNA gene copies per PCR reaction were created by performing 10-fold serial dilutions and were tested in duplicate on each qPCR plate. PCR efficiencies ranged from 1.9 to 2.1 for all plates.

### 2.6 Microbial community analysis

DNA was sent to the Environmental Sample Preparation and Sequencing Facility at Argonne National Laboratory (Lemont, IL) for amplicon sequencing using the 515F-806R primers (Apprill et al., 2015; Parada et al., 2016) for the V4 region of the 16S rRNA gene to profile archaeal and bacterial communities. PCR amplicon libraries targeting the 16S rRNA encoding gene were created using a barcoded primer set adapted for the Illumina MiSeq (Caporaso et al., 2012). Each 25 μL PCR reaction contained 9.5 μL Certified DNA-Free water, 12.5 μL of QuantaBio’s AccuStart II PCR ToughMix (2x concentration, 1x final), 1 μL Golay barcode tagged Forward Primer (5 μM concentration, 200 pM final), 1 μL Reverse Primer (5 μM concentration, 200 pM final), and 1 μL of template DNA. The PCR conditions were: 94°C for 3 min to denature the DNA, with 35 cycles at 94°C for 45 s, 50°C for 60 s, and 72°C for 90 s; with a final extension of 10 min at 72°C to ensure complete amplification. Samples were sequenced on the Illumina MiSeq with 2 × 250 bp reads. Extraction blanks were also included as controls for DNA extraction and sequencing.

Raw sequencing reads were processed with the dada2 v1.18.0 pipeline in R v4.3.2 (Callahan et al., 2016; R Core Team, 2018) to identify amplicon sequence variants (ASVs) (Callahan et al., 2017). Code for data processing is available from GitHub [adapted from Baker (2023)]. Sequences were demultiplexed and fastq files were assessed for quality using FastQC v0.11.9 (Brown et al., 2017). Sequences were processed to remove ambiguous bases and primers were removed using cutadapt v4.6 (Martin, 2011). Reads were truncated at a quality score of 2 and filtered to remove reads with expected errors of > 2. Error profiles were estimated using 1e8 bases and sequences were inferred using data 2. Paired end reads were merged and samples were combined into a sequence table. Chimeras were removed using the consensus method. Taxonomy was assigned using the SILVA database v138.1 (Quast et al., 2012). ASVs identified as mitochondria and chloroplasts were removed from the dataset. Results were exported into a phyloseq object for downstream statistical analysis (McMurdie and Holmes, 2013).

Shared ASVs between the origin soil layers (100% AL-t0 and 100% PF-t0) and the post-incubation samples (100% AL-tf and 100% PF-tf) were compared within each site to assess the effects of soil warming for 100 days on ASV turnover. ASV overlap between the experimentally mixed samples and the unmixed soil layers was also assessed by comparing the ASVs present in the mixed samples to the origin (t0) and post-incubation (tf) samples. ASV overlap was not compared across experimentally mixed samples. First, a list of ASVs with greater than 0 abundance in at least one replicate was generated for each treatment. ASVs unique to each treatment were filtered and counted. ASV overlap was determined by taking the intersect of ASVs for each treatment comparison.

### 2.7 Statistical analysis

Statistical analyses were performed in R. One-way analysis of variance (ANOVA) and *t*-test were used to determine significant differences in abiotic and biotic variables of the samples within sites. We performed a Welch two-sample *t*-test to estimate mean differences in soil abiotic properties between APT and TK1 sites for the active layer, transition zone and permafrost to evaluate the intrinsic differences between both sampling sites. One-way analysis of variance (ANOVA) was used to assess differences in various dependent variables (soil properties, respiration rate, AWCD, SWCD, bacterial gene copy numbers, and alpha diversity of substrate utilization and microbial communities) across mix ratios within each site. Assumptions of normality were assessed using the Shapiro–Wilk test and homogeneity was assessed using the Levene test. Multiple comparison was conducted using the Tukey’s HSD test to determine significant differences between means across treatments. Bacterial gene abundance t0 samples required log transformation to meet the assumptions of the ANOVA.

Analysis of the microbial community data was performed using the “phyloseq” (McMurdie and Holmes, 2013) and “vegan” (Oksanen et al., 2020) packages in R. Sequences were rarified to a depth of 10,000 reads per sample (Supplementary Figure 1). The Shannon diversity index was used to assess differences in alpha diversity across mix ratios for both sites. Non-parametric multidimensional scaling (NMDS) using Bray-Curtis dissimilarity measure was used to visualize differences in microbial community composition when soils were experimentally mixed. Permutational multivariate analysis of variance (PERMANOVA) was conducted to identify if microbial communities were significantly different by mix ratio.

## 3 Results

### 3.1 Abiotic soil properties

**FIGURE 1:**
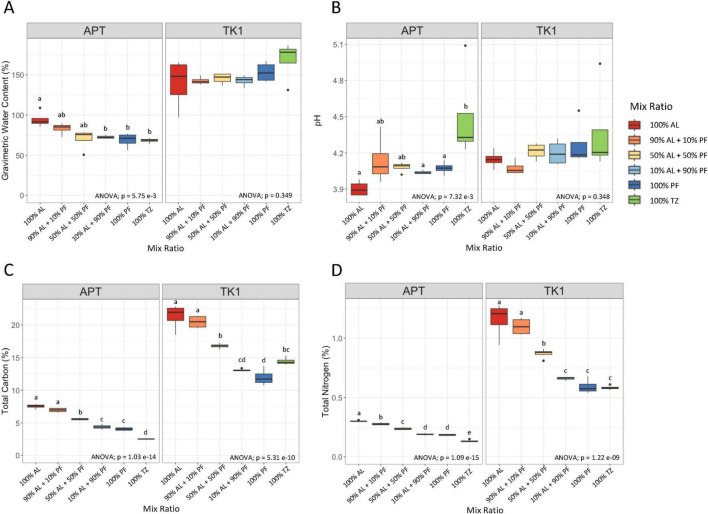
Abiotic soil properties of **(A)** gravimetric water content, **(B)** pH, **(C)** total carbon, and **(D)** total nitrogen of mixed and unmixed soils at the conclusion of the soil incubation. Boxplots show median value (*n* = 4) as a solid line and upper and lower quartiles at the top and bottom of the boxes, respectively. Whiskers and points indicate the extent of the data. One-way ANOVA at the α = 0.05 significance level was conducted to examine differences in soil properties across mix ratios. Different letters above the boxes indicate significant differences in the means determined by the Tukey HSD *post hoc* test.

Abiotic soil properties of the AL, TZ, and PF differed between the two locations where samples were collected for this laboratory experiment (Supplementary Table 1). Significant differences between soil properties were also observed when comparing the unmixed soil layers within each site (Figure 1). In general, the 100% AL samples were more water saturated and had higher carbon and nitrogen contents compared to the 100% PF and 100% TZ for both sites. The 100% AL was slightly more acidic than the TZ at the APT site. Abiotic soil properties measured at the conclusion of the incubation were affected by the mixing of AL and PF soils (Figure 1). In the APT samples, gravimetric water content, total carbon, and total nitrogen decreased as the proportion of PF increased. A similar trend was observed for the total carbon and nitrogen in the TK1 samples. GWC was not significantly different between the mix ratios of the TK1 samples. Soil pH was similar across all mix ratios for both sites. Linear regression of soil property measurements by proportion of PF in the mixed samples showed strong negative trends for total carbon (APT R^2^ = 0.9592, TK1 R^2^ = 0.9333), total nitrogen (APT R^2^ = 0.9688, TK1 R^2^ = 0.9098) and GWC for the APT site (R^2^ = 0.4464). The TZ had lower total carbon and nitrogen compared to PF in the APT samples, however it was similar or slightly higher than PF in the TK1 samples.

### 3.2 Microbial respiration

**FIGURE 2:**
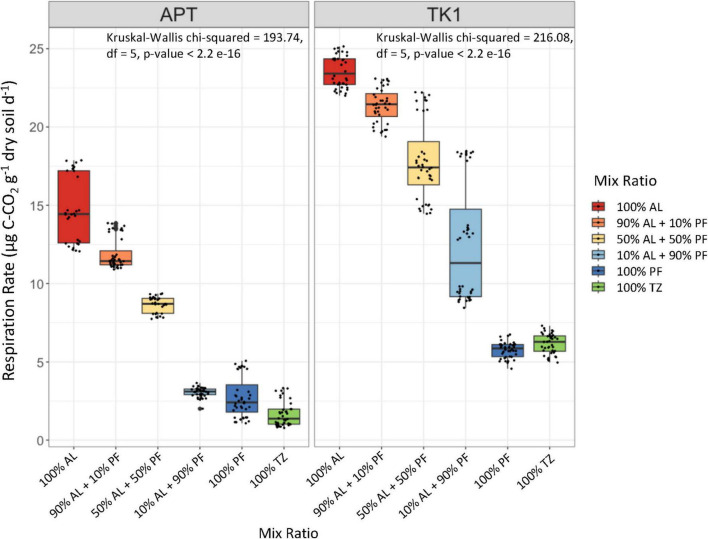
Stabilized heterotrophic respiration rates over the last 108 h of incubation at 10°C. Boxplots show median value (*n* = 80) as a solid line and upper and lower quartiles at the top and bottom of the boxes, respectively. Whiskers and points indicate the extent of the data. Jitters represent respiration rate observations from every 12 h over the last 108 h of incubation. The Kruskal-Wallis test was performed to assess significant difference in respiration rates because the assumption of normality was not met.

Heterotrophic respiration rates were continuously measured over the course of the 100 days incubation. The stabilized respiration rates (slope < 0.005) over the last 108 h of incubation were used to assess significant differences in microbial activity across the mixed samples. Respiration rates were highest in the 100% AL samples and decreased significantly (*p* < 2.2 e-16) as the proportion of PF in the microcosm increased (Figure 2). Mean respiration rates were negatively correlated with the proportion of PF in the sample (APT R^2^ = 0.9815, TK1 R^2^ = 0.9164). The 100% TZ samples had similar respirations rates compared to the 100% PF samples within each site. The TK1 samples were on average 9 μg C-CO_2_ g^–1^ dry soil d^–1^ higher than the APT site when comparing mix ratios between the sites, except for the 100% PF and 100% TZ samples where were approximately 3 and 4.5 μg C-CO_2_ g^–1^ dry soil d^–1^ higher, respectively.

### 3.3 Carbon substrate utilization

**FIGURE 3:**
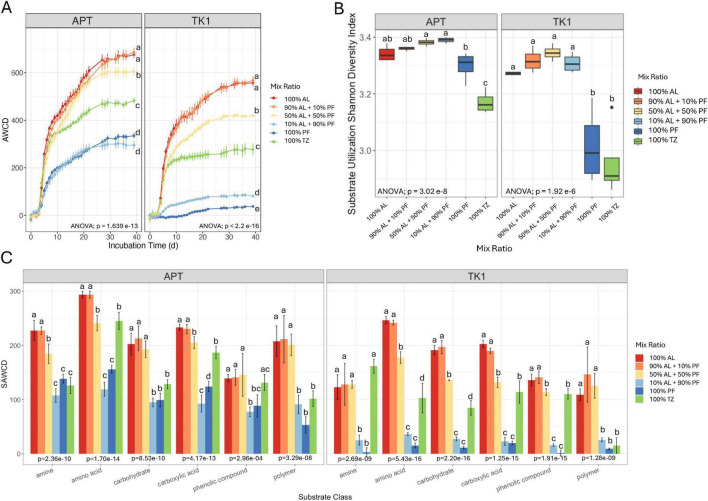
Microbial substrate utilization activity as measured by the Biolog EcoPlates™ at the end of the soil incubation with **(A)** average well color development (ACWD) over time, **(B)** substrate utilization Shannon diversity index and **(C)** substrate-specific AWCD (SAWCD) at day 39. Vertical lines on the line and bar charts indicate standard error of the mean (*n* = 4). Boxplots show median value as a solid line and upper and lower quartiles at the top and bottom of the boxes, respectively. Whiskers and points indicate the extent of the data. One-way ANOVA at the α = 0.05 significance level was conducted to examine differences in AWCD, alpha diversity, and SAWCD across mix ratios. Different letters above the boxes indicate significant differences in the means determined by the Tukey HSD *post hoc* test.

Microbial community carbon substrate utilization was assessed using the Biolog EcoPlates™. There were significant differences in AWCD across the mix ratios at the final sampling interval (APT *p* = 1.639 e-13; TK1 *p* < 2.2 e-16). Average well color development (AWCD) was highest in the 100% AL and 90% AL + 10% PF, followed by the 50% AL + 50% PF then 100% TZ for both sites (Figure 3A). The APT 10% AL + 90% PF and 100% PF samples had similar AWCD at the final measurement. For the TK1 site, the AWCD from 100% PF was lower than the 10% AL + 90% PF samples.

Carbon substrate utilization alpha diversity was highest in the samples where AL soil was present (Figure 3B). The addition of 10% PF–90% AL did not change the substrate use diversity compared to AL alone. However, adding a small amount of AL to PF (i.e., 10% AL + 90% PF) significantly increased substrate utilization diversity at both sites. The 50% AL + 50% PF samples showed slightly higher substrate diversity compared to AL alone, although this difference was not significant. The 100% TZ samples had lower substrate utilization compared to all other soils within each site, except for the 100% PF TK1 samples.

Mixing AL and PF soils had a significant (*p* < 1 e-3) effect on the degree to which the microbial community utilized substrates within a given substrate class (Figure 3C). The 100% AL and 100% PF SAWCD were significantly different across all substrate classes in both locations. In general, 100% AL and 90% AL + 10% PF had the highest utilization across the substrate classes and were similar to one another when comparing within each substrate class for each site. The addition of 10% AL had no significant effect on substrate utilization when compared to 100% PF except for carboxylic acids in the APT samples. The SAWCDs for the 10% AL + 90% PF and 100% PF samples were substantially lower than the other samples for TK1 site, however substrate utilization diversity (Figure 3B) was similar to all samples that contained AL.

### 3.4 Effect of mixing on bacterial communities

**FIGURE 4:**
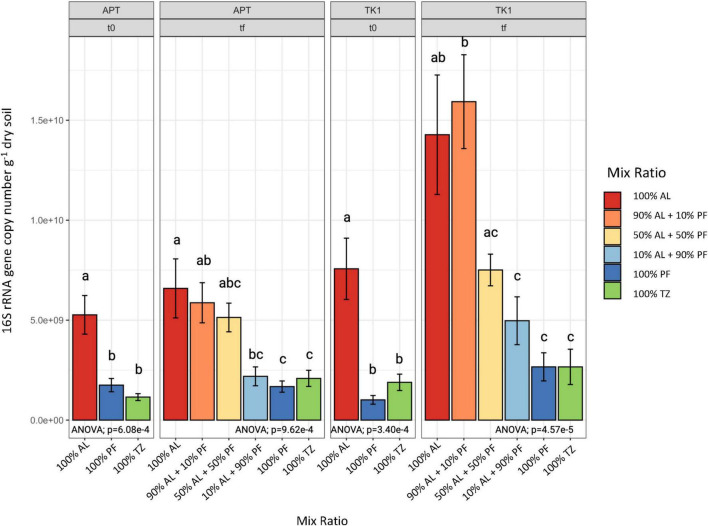
Bacterial abundance measure by 16S rRNA gene copy number per gram of dry soil. Vertical lines on the bar chart indicate standard error of the mean (*n* = 4). One-way ANOVA at the α = 0.05 significance level was conducted to examine differences in gene abundance across soil layers and mix ratios. Different letters above the boxes indicate significant differences in the means determined by the Tukey HSD *post hoc* test. T0 samples were log transformed to meet the assumptions of the ANOVA.

At both the beginning and end of the soil incubation, the AL samples had significantly higher bacterial gene abundance per gram of dry soil compared to TZ and PF soils as measured by qPCR (Figure 4). The 100% AL and 90% AL + 10% PF samples were similar in bacterial gene abundance, and for the APT site, the 50% AL + 50% PF was also similar (Figure 4). Although a decreasing trend was observed as the amount of AL was decreased from 90% to 10%, the observed change was only significant between the 90% AL + 10% PF and those with 50% PF or 10% PF in the TK1 samples. Within each site, samples that contained 50%–100% PF as well as the 100% TZ all had similar bacterial gene abundance (Figure 4).

**FIGURE 5:**
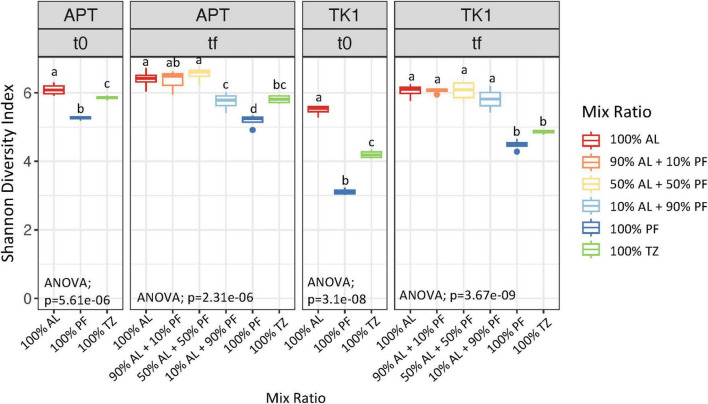
Microbial community alpha diversity measured by Shannon diversity index for bacterial communities. Boxplots show median value as a solid line and upper and lower quartiles at the top and bottom of the boxes, respectively. Whiskers and points indicate the extent of the data. One-way ANOVA at the α = 0.05 significance level was conducted to examine differences in microbial diversity across mix ratios. Different letters above the boxes indicate significant differences in the means determined by the Tukey HSD *post hoc* test.

Amplicon sequencing analysis of alpha diversity showed significantly different diversity at the start of the incubation (*p* = 5.61 e-06) across the AL, TZ, and PF samples at both sites (Figure 5). Alpha diversity was highest in the AL soils followed by the TZ then PF samples (Figure 5). Post incubation samples from the APT site showed similar alpha diversity between 100% AL, 90% AL + 10% PF, and 50% AL + 50% PF samples. The 10% AL + 90% PF samples were significantly lower than those with mixes containing higher percentages of the AL soil, but similar to those of the 100% TZ. The 100% PF samples had the lowest alpha diversity throughout the experiment (Figure 5). The TK1 samples showed similar alpha diversity in all samples that contained AL soil. The 100% TZ and 100% PF samples had similar alpha diversity. The majority of the dominant taxa across both sites were members of the phyla Pseudomonadota, Acidobacteriota, Actinomycetota, and Verrucomicrobiota (Supplementary Figure 2). See Supplementary materials for bar charts at order (Supplementary Figure 3), family (Supplementary Figure 4), and genus (Supplementary Figure 5) taxonomic resolutions. Archaea were present in relative abundances less than 0.05% with the APT-100% PF containing the greatest proportion (Supplementary Figure 6). Members of the genera *Methanobacterium* and *Methanosarcina*, both known as anaerobic methane producing archaea, were most abundant across samples even after aerobic incubation, however, we cannot say if these organisms were active given DNA sequencing was used.

**FIGURE 6:**
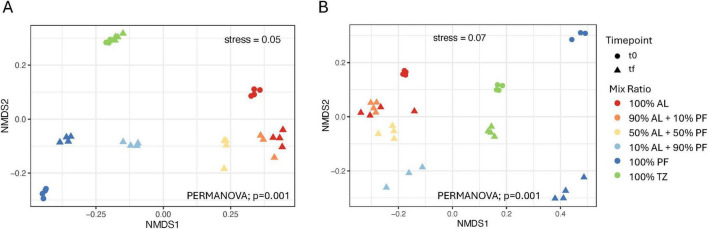
Non-metric multidimensional scaling (NMDS) analysis of **(A)** APT and **(B)** TK1 bacterial community composition using Bray–Curtis dissimilarity. PERMANOVA was used to determine significant difference in community composition by mix ratio.

Significant differences in beta diversity across mix ratios were observed for both the APT (*p* = 0.001) and TK1 (*p* = 0.001) site (Figures 6A, B). For both sites, bacterial communities from the start of the incubation formed distinct, but soil layer specific, clusters. The 100% AL and 100% PF communities shifted over the course of the 100 days soil incubation for both sites. For both sites at the end of the incubation, samples clusterd based on the increasing amount of AL soil added. There were similar bacterial communities between the 100% AL and 90% AL + 10% PF samples. The 50% AL + 50% PF samples were more like those with a greater proportion of AL soil compared to PF dominated samples. The 10% AL + 90% PF clustered separately from the other mix ratios that were mostly clustered near the 100% AL samples. The separation of 10% AL + 90% PF and the other samples containing AL along NMDS1 was greater for the APT site compared to TK1. The 100% TZ samples clustered separately as well and were dissimilar to the experimentally mixed samples and 100% PF.

**FIGURE 7:**
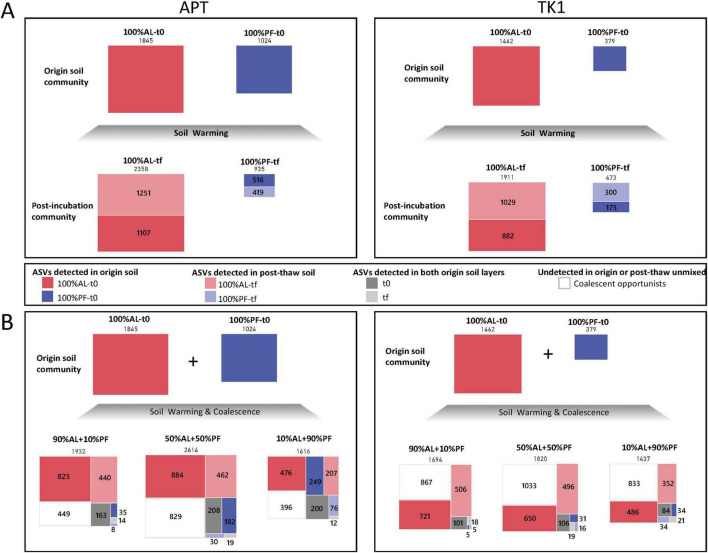
Distribution of community members by soil origin for **(A)** pre- and post-incubation at 10°C and **(B)** before and after experimental mixing. The numbers above the boxes indicate total amplicon sequence variants (ASVs) across microcosms of that type (*n* = 4). The numbers inside or next to the boxes indicate the number of ASVs shared with the origin soil (dark colors) or the post-thaw unmixed samples (light colors). ASVs shared with active layer (AL) soils are in red and ASVs shared with permafrost (PF) soils are in blue. ASVs in gray were shared with both the AL and PF soil layers and their origin could not be discerned. ASVs in white were not detected in either the origin (t0) or post-thaw (tf). Boxes are proportional based on total ASV count.

The degree of ASV overlap between the unmixed soil layers and experimentally mixed samples was assessed to determine the effects of soil warming and coalescence on ASV turnover (Figure 7). For both sites, the 100 days soil incubation resulted in an increase in the 100% AL total richness, but a decrease in the amount of ASVs detectable at the start of the incubation (Figure 7A). The 100% PF samples also showed a decrease in ASVs detected at the start of the incubation but an increase in overall ASV richness for TK1 PF samples. When comparing the experimentally mixed samples to the pre- and post-incubation origin soils, there was a large proportion of the mixed communities that were undetected in either origin soil (Figure 7B).

## 4 Discussion

### 4.1 Active layer soils have greater microbial activity and diversity compared to permafrost

At both sites, the AL soil had significantly higher microbial activity and functional diversity compared to the TZ and PF even though the soil properties and starting microbial community structure were different between the two sites. Specifically, heterotrophic respiration was four to five times higher in the 100% AL samples compared to 100% PF, with the 100% TZ behaving similarly to the PF samples. The physical mixing of the soils would have introduced oxygen into the soil pore space and likely activated heterotrophic carbon consumption. Our results support previous findings that AL microbial communities generally have faster growth rates and higher respiration compared to the TZ or PF (Ernakovich and Wallenstein, 2015; Monteux et al., 2018; Doherty et al., 2020).

Carbon utilization as measured by AWCD was significantly higher in the AL soils, as compared to the TZ and PF soils. Only the TK1 site showed a significant difference in carbon utilization diversity between AL and PF samples (Figure 3B). PF microbial communities utilize fewer carbon substrates and have slower growth rates compared to AL soil likely due to lower taxonomic diversity or greater chemical complexity of organic matter found in PF (Ernakovich and Wallenstein, 2015). In our study, AL samples had more C and N content resulting in greater nutrient availability for utilization by the microbial communities. The rapid PF thaw of this experiment may have also contributed to community instability through rapid environmental change and therefore lowered functional output of the PF communities (De Vries and Shade, 2013).

Bacterial gene abundance and taxonomic diversity were higher in the AL soils compared to PF. We chose to focus on molecular approaches that profile prokaryotic community members which potentially limits our understanding of whole community dynamics since other microorganisms such as fungi, algae, and protozoa likely contributed to microbial activity and carbon utilization. Previous studies have shown more taxonomically diverse microbial communities in AL soils compared to PF (Ernakovich and Wallenstein, 2015; Hultman et al., 2015; Tripathi et al., 2018; Wu et al., 2021). Increased nutrient availability (Reyes and Lougheed, 2015), warmer soil temperatures (Douglas et al., 2021; Baker et al., 2023), and active dispersal (Doherty et al., 2020; Ernakovich et al., 2022) in the AL likely contribute to the higher diversity of microbial communities compared to PF. These two soil layers set the environment and seeded the microbial communities for the mixing experiment.

### 4.2 Microbial community coalescence varied depending on the proportion of AL to PF

In permafrost-affected environments, it is becoming more likely that these distinct habitats and communities will coalesce upon thaw, forming mixed microbiomes experiencing new environmental conditions and possibly changing the community structure and function in these systems (Ernakovich et al., 2022). It is critical to consider these coalescence events in order to predict microbiome shifts due to thaw disturbance and the effects on ecosystems. In nature, most coalescence events occur in unequal mixing ratios (Rillig et al., 2015; Mansour et al., 2018; Qiao et al., 2024) and this is expected with different PF thaw conditions. For instance, the 90% + 10% samples likely best represented AL deepening where thawed PF will gradually transition from PF to TZ to AL. The 50% AL + 50% PF was more representative of rapid thaw, or thermokarst, where the thawed environment is likely compositionally different from a gradual thaw scenario. Our results indicate the degree of soil mixing (i.e., proportion of AL versus PF in the final mixed community) drives microbial community dynamics.

Diversity and abundance of the AL and PF origin communities may have influenced the outcomes of community coalescence. Greater bacterial abundance in the AL likely resulted in communities closely resembling those of the 100% AL samples even when 50% of the mix was comprised of PF. Higher bacterial gene abundance and more microbial activity may explain why the TK1 10% AL + 90% PF mix was more similar in community composition to other samples with AL soil present than what was observed for the APT site. Furthermore, The TK1 site had a greater difference in community diversity between AL and PF samples at the time of mixing. Dominant species can influence the abundance of other taxa during coalescence through both predation or commensal mechanisms such as metabolic secretions (Qiao et al., 2024). More work is needed to understand the active role dominant taxa played in shaping the mixed microbiomes.

Taxa emerged that were previously undetected in either origin soil (AL or PF at t0) when soils were warmed and when AL and PF soils were mixed. Soil warming alone increased the overall community richness of the 100% AL samples at both sites, however, there was a decrease in the number of ASVs that were previously detected at the start of the incubation and an emergence of those that were originally undetected. This was also observed for the 100% PF samples, however community richness decreased for the APT site after warming. These results suggest that soil warming can have both a positive and negative effect on Arctic soil microbiota where some taxa become undetectable while others become more abundant. Soil coalescence in the experimentally mixed samples also resulted in a large proportion of previously undetected taxa emergence. Previous work in marine environments showed that communities with higher diversity do not necessarily drive community structure during coalescence events and that initially rare taxa become more abundant in the mixed community (Rocca et al., 2020). Although we also observed an emergence of rare taxa with coalescence of AL and PF, soil environments are arguably more complex and heterogeneous than aqueous environments which may explain why starting community diversity also influenced community coalescence events in our experiment. We suspect the previously undetected members of the thawed and mixed samples were present in the origin soil layers but were in a dormant state that influenced our ability to extract DNA or that we did not capture them with the sampling depth of our sequencing.

Differences between the soil properties of the starting soil layers (i.e., AL and PF) likely influenced community coalescence upon mixing. In general, the APT site had more similar soil properties between the 100% AL and 100% PF samples compared to those of the TK1 site. The more similar the home environments of the mixed samples may result in less selective filters and species sorting but more biotic interactions influencing community coalescence due to niche overlap (Rocca et al., 2020). This could give more opportunity for a new community to assemble given fewer selective filters rather than a community that closely resembles the dominant soil type as seen in the APT 10% AL + 90% PF mix. Strong asymmetric environmental filtering or community resistance could explain why the 50% AL + 50% PF and 90% AL + 10% PF communities closely resembled those of the 100% AL (Rocca et al., 2020). The greater difference in soil properties of the origin soil layers of the TK1 site may have contributed to the large number of previously undetected taxa that emerged with coalescence. The new abiotic environment of the mixed soils could have resulted in new niches for the dormant or rare taxa to reproduce to the point they were now detectable post-incubation.

### 4.3 Active layer–permafrost soil mixing increased functional activity compared to permafrost alone

In the experimentally mixed samples, we saw that a greater proportion of AL soil resulted in significant increases in functional activity and diversity after 100 days of incubation. Heterotrophic respiration rates were highest in the 100% AL samples and decreased as the proportion of PF increased, possibly due to more carbon availability in samples with more AL soil present. There was also a general increase in copiotrophic taxa of the Pseudomonadota and Bacteroidota phyla when soils were warmed as well as with increasing proportion of PF in the mixed samples. The relative abundances of oligotrophic taxa of the Acidobacteriota, Verrucomicrobiota, and Planctomycetota phyla decreased with greater proportion of PF in the sample. There was an increase in Actinomycetota with more permafrost in the mixed samples, however, this phylum contains both oligotrophic and copiotrophic organisms (Fierer et al., 2007). Previous studies have also observed an increase in copiotrophic taxa and a decrease in oligotrophic community members when AL and PF soils were warmed separately (Schostag et al., 2019; Perez-Mon et al., 2022; Scheel et al., 2022, 2023). Such findings were attributed to carbon or nitrogen limitation in AL soil and that PF thaw may benefit microbial taxa by releasing substrates (Perez-Mon et al., 2022; Sipes et al., 2022). Our results suggest that as permafrost thaws and soils mix, previously inaccessible carbon will become available to PF communities and AL communities as they are dispersed resulting in more CO_2_ released from soils compared to estimates derived from incubations where PF was thawed alone.

The addition of just 10% AL significantly increased substrate utilization diversity compared to PF alone, however, substrate specific activities were slightly lower in the mixed samples. This suggests that mixing results in a broader suite of carbon substrates that can be utilized by the microbial community, but at lower rates compared to PF alone, possible due to competition within the community for these resources. Additionally, mixes that contained a greater proportion of permafrost may have fewer or different nutrient sources available to the AL community when introduced. Previous studies have shown that microbial coalescence restores soil function and are dominated by communities that use resources efficiently (Sierocinski et al., 2017; Huet et al., 2023). The PF community may be contributing unique traits to the mixed community which is making a significant effect on function even in a small population size. The emergence of previously rare taxa may have also contributed to increased functional potential compared to PF alone as these taxa may bring new functions. Microbial communities that can use a greater diversity of carbon substrates are likely more effective in decomposition and nutrient cycling since they can break down complex carbon sources. Our results support previous work that showed PF functional limitations can be alleviated with the addition of more diverse microbial communities (Monteux et al., 2020) and greater nutrient content, such as those found in the AL soil. Mixed AL-PF communities used more diverse carbon substrates, which could make them more adaptable or resilient to changing environmental conditions and also influence higher organisms like plant growth (Qiao et al., 2024).

### 4.4 Experimental mixing does not reflect *in situ* transition zone

The TZ is the boundary layer where AL and PF meet and the soil that has most recently turned from PF to AL in regions undergoing PF thaw. This is where we would expect natural community coalescence to occur in field settings. Our results indicate that our experimentally mixed soils did not accurately represent the *in situ* microbial dynamics in the TZ. Community structure and substrate utilization was significantly different between the TZ and experimentally mixed samples and soil activity was more similar to the PF samples. The TZ is a unique area along the soil column that represents the summertime thaw boundary where metals such as iron and other elements can accumulate during the growing season (Ji et al., 2021; Barker et al., 2023). Interestingly, the 100% TZ samples had higher bacterial alpha diversity compared to 100% PF but showed lower substrate use diversity for the APT site, suggesting a higher degree of functional redundancy at the TZ. Functional redundancy can lead to ecosystem stability because if a species group is lost, the function still occurs (Biggs et al., 2020). Conversely, the PF communities had less taxonomic diversity but greater functional diversity, indicating that a few species are performing many functions. Therefore, if a species or group of microorganisms are lost, then the ecosystem process may not occur. Lower functional redundancy in permafrost microbiomes increases the uncertainty in stochastic community assembly during thaw and since functions may be lost with community turnover (Ernakovich et al., 2022). Permafrost thaw can occur quickly or slowly depending on site location and thaw conditions. Our lab incubation estimated the maximum interaction between AL and PF by directly mixing these samples. Soil mixing is a major disturbance that may not reflect microbial dynamics at the TZ where PF is more slowly transitioned to seasonally-thawed AL.

## 5 Conclusion

Active layer and PF microbiome communities will mix as permafrost continues to rapidly thaw. Our results demonstrate the importance of AL microbial community contributions to thawed PF. Our hypothesis was supported in that the mixing of AL soil and PF resulted in carbon utilization increases compared to PF alone, but when PF was added to AL soil, there was little effect on carbon utilization because AL soils contained greater biomass and microbial diversity compared to PF. These results suggest that when the proportion of AL is higher in the mixed soil, the entire sample is more reflective of the legacy AL even though the starting communities between AL and PL appear different. The AL soil will contribute significant changes in microbial community composition and function as it mixes with newly thawed PF such as more carbon released from soil as CO_2_ if mixed communities are dominated by copiotrophic taxa that release more carbon. The broader functional potential and ability to decompose soil organic matter when AL and PF mix could significantly increase carbon feedbacks that are currently unaccounted for in global climate models.

## Data Availability

The datasets presented in this study can be found in online repositories. The names of the repository/repositories and accession number(s) can be found below: https://www.ncbi.nlm.nih.gov/, BioProject PRJNA1173724. The code used for sequencing and EcoPlate™ analyses is available at GitHub (https://github.com/sljarvis2/al_pf_mix_public).
