# Supplementary material for: Active layer and permafrost microbial community coalescence increases soil activity and diversity in mixed communities compared to permafrost alone
Source: Front Microbiol. 2025 Jun 5;16:1579156. doi: 10.3389/fmicb.2025.1579156 (PMC12178574; doi:10.3389/fmicb.2025.1579156)
Supplement: Supplementary file 1 [file Data_Sheet_1.docx]

Supplementary Material

# Supplementary Tables

Table S1. Abiotic soil properties of the active layer (AL), transition zone (TZ), and permafrost (PF) soil layers. Gravimetric water content (GWC %), soil pH, total carbon (C %), and total nitrogen (N %) were measured at end of the incubation. Means and standard error are shown (n = 4). Significant differences in means between sites were assessed with the Welch t-test.

|  |  |  |  | | |
| --- | --- | --- | --- | --- | --- |
| Soil Layer | Site | GWC % | pH | C % | N % |
| AL | APT | 94.36±5.04 | 3.90±0.032 | 7.53±0.18 | 0.30±0.0025 |
|  | TK1 | 139.75±15.74 | 4.15±0.037 | 21.31±0.98 | 1.16±0.075 |
|  | p-value | 0.0578 | **2.54E-03** | **5.89E-04** | **1.43E-03** |
| TZ | APT | 68.44±1.52 | 4.50±0.20 | 2.53±0.033 | 0.14±0.0050 |
|  | TK1 | 168.44±12.62 | 4.37±0.19 | 14.48±0.30 | 0.58±0.010 |
|  | p-value | **3.86E-03** | 0.6669 | **2.92E-05** | **1.08E-06** |
| PF | APT | 69.04±4.60 | 4.075±0.027 | 4.07±0.13 | 0.19±0.0029 |
|  | TK1 | 153.50±6.32 | 4.27±0.094 | 12.00±0.65 | 0.60±0.031 |
|  | p-value | **6.70E-05** | 0.1264 | **8.22E-04** | **8.76E-04** |

# Supplementary Figures

#

# Supplementary Figure 1. (A) Sequencing reads per sample where “NA” indicates extraction blanks and (B) rarefaction curves for 10,000 reads and (C) all reads.

**Supplementary Figure 2.** Taxa bar chart of the top 10 phyla in the data for APT and TK1 samples pre- (t0) and post-incubation (tf).

**Supplementary Figure 3.** Taxa bar chart of the top 10 orders in the data for APT and TK1 samples pre- (t0) and post-incubation (tf).

**Supplementary Figure 4.** Taxa bar chart of the top 10 families in the data for APT and TK1 samples pre- (t0) and post-incubation (tf).

**Supplementary Figure 5.** Taxa bar chart of the top 10 genera in the data for APT and TK1 samples pre- (t0) and post-incubation (tf).

**Supplementary Figure 6.** Taxa bar chart of archaea genera present in the 16S rRNA dataset in the data for APT and TK1 samples pre- (t0) and post-incubation (tf).
